# Acute Kidney Injury and Blood Purification Techniques in Severe COVID-19 Patients

**DOI:** 10.3390/jcm11216286

**Published:** 2022-10-25

**Authors:** Marianna Napoli, Michele Provenzano, Lilio Hu, Claudia Bini, Chiara Abenavoli, Gaetano La Manna, Giorgia Comai

**Affiliations:** 1 Nephrology, Dialysis and Renal Transplant Unit, IRCCS-Azienda Ospedaliero-Universitaria di Bologna, Via Massarenti 9, 40138 Bologna, Italy; 2 Alma Mater Studiorum, University of Bologna, 40138 Bologna, Italy

**Keywords:** SARS-CoV-2, prognosis, hemodialysis, AKI, kidney disease

## Abstract

Although most patients with severe acute respiratory syndrome coronavirus-2 (SARS-CoV-2) experience respiratory manifestations, multi-organ dysfunction is frequent. Almost 20% of hospitalized patients with SARS-CoV-2 infection develop acute kidney injury (AKI). The pathophysiology of AKI is a result of both the direct and indirect effects of SARS-CoV-2 infection, including systemic inflammatory responses, the activation of the renin-angiotensin-aldosterone system (RAAS), and endothelial and coagulative dysfunction. Underlying SARS-CoV-2 infection-associated AKI, an immunological hyper-response with an unbalanced innate and adaptative response defined as a “cytokine storm” has emerged. Numerous agents have been tested in an effort to mitigate the cytokine storm, and a range of extracorporeal cytokine removal techniques have been proposed as potential therapeutic options. In the present review, we summarize the main pathogenetic mechanisms underlying COVID-19-related AKI in order to provide an appropriate individual therapeutic strategy to improve clinical outcomes and limit the progression of early disease.

## 1. Introduction

The severe acute respiratory syndrome coronavirus-2 (SARS-CoV-2) disease, also known by the acronym “COVID-19” (Coronavirus Disease 19), was recognized as an international health emergency by the World Health Organization (WHO) on 11 March 2020 [1].

COVID-19 primarily manifests with respiratory distress, but it may involve multiple organ systems, including the kidneys, with the onset of acute kidney injury (AKI), hematuria, and proteinuria, together with histopathological signs of kidney damage [2]. In a large observational study of hospitalized patients with COVID-19, AKI occurred in 46% of patients with variable severity (the prevalence of disease in stages 1, 2, and 3 was 39%, 19%, and 42%, respectively) [3]. AKI is defined according to the Kidney Disease Improving Global Outcomes Work Group (KDIGO) criteria. It is detected in up to 45% of COVID-19 patients in intensive care units (ICUs) requiring kidney replacement therapy [4,5,6,7], and is strictly associated with disease severity and mortality [8,9]. Age and pre-existing comorbidities, such as a history of diabetes mellitus, chronic kidney disease (CKD), and cardiovascular (CV) disease, raise the risk of AKI. Moreover, CKD is an independent risk factor for the development of severe forms of COVID-19, and it is associated with worse future outcomes, including pulmonary infection; all-cause death [10,11,12,13]. The high risk of AKI among patients with COVID-19 stimulates a better comprehension of the mechanisms underlying kidney damage with the aim of establishing the appropriate individual treatment and improving prognosis in these frail patients.

Among COVID-19 patients, acute tubular injury (ATI) is the most frequent histological finding detectable in the kidney biopsy [14,15], associated with local and systemic inflammatory responses, the activation of the renin-angiotensin-aldosterone system (RAAS), and endothelial and coagulative dysfunction. The balance between pro- and anti-inflammatory mediators in a cytokine storm syndrome seems to have a pivotal role in determining the degree of COVID-19 severity [16,17].

Extracorporeal (EC) procedures, such as continuous renal replacement therapy (CRRT) and immunoadsorption, have been proposed in some available guidelines as a therapeutic strategy to reduce inflammation in patients with COVID-19 and AKI [18]. However, although these extracorporeal cytokine removal techniques were attractive and potentially helpful in this setting of patients, their efficacy on significant clinical outcomes was not proven [19,20].

The aim of the present narrative review is to summarize the principal mechanisms underlying inflammation in patients with concomitant COVID-19 and AKI and how these patterns can be modified by EC procedures.

## 2. Pathophysiology of AKI in COVID-19: Molecular Pathways

**Figure 1:**
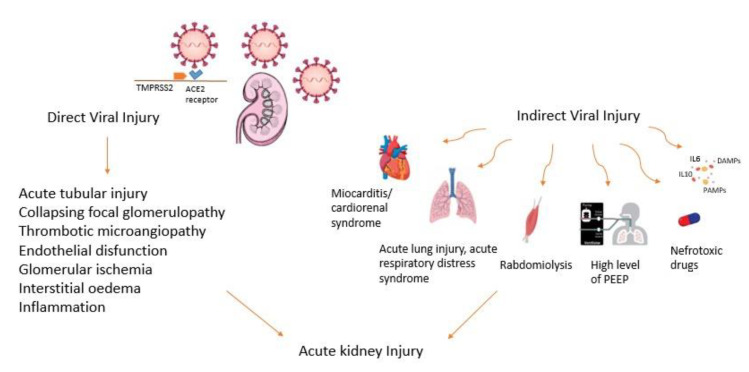
Direct and indirect effects of SARS-CoV-2 infection on the pathophysiology of AKI.

The pathophysiology of AKI in COVID-19 patients is a result of both the direct and indirect effects of SARS-CoV-2 infection (Figure 1) [21,22]. The hypothesis that the virus directly affects and damages renal tissue is supported by the finding of SARS-CoV-2 protein in autopsy kidney tissue of patients who likely died because of COVID-19 [23,24,25,26].

Several indirect effects have been proposed, including the overlap of decreased cardiac output in cardiorenal syndrome with hypoxia, renal hypotension or renal vein congestion (for example, during cardiomyopathy or acute viral myocarditis), toxic injury with rhabdomyolysis, or the effect of nephrotoxins like vancomycin, aminoglycosides, and colistine [27]. The reduced renal flow induced by sedation and the high positive end-expiratory pressure applied by mechanical ventilation in ICU impact negatively on cardiac output and contribute to kidney damage with the onset of cardio-renal syndrome type I [27,28,29].

Additional indirect causes of AKI that have been proposed include a ‘’cytokine storm’’, endothelial injury, activation and dysregulation of coagulation, and alterations of RAAS pathways [6,29,30].

Extracorporeal membrane oxygenation (ECMO) and CRRT techniques can also contribute to the deleterious effects of cytokines [31]. The production of cytokines by peripheral blood mononuclear cells (including monocytes, T-lymphocytes, and natural killer cells) is induced by the passage of endotoxin fragments from a contaminated dialysate to the blood through the dialytic membrane by complement activation due to blood-membrane interactions and by physical contact of monocytes to the filter’s cellulosic membranes.

### 2.1. The Role of ACE

Infection with SARS-CoV-2 is a multistep process [32]. SARS-CoV-2 enters the human body through respiratory droplets [33] using surface S protein which is composed of S1 and S2 subunits. The S1 subunit interacts with Angiotensin Converting Enzyme-2 (ACE-2) and then with transmembrane serine protease 2 (TMPRSS2), leading to the proteolytic cleavage and conformational change of S protein and endocytosis in the host cell [34,35,36,37].

ACE-2 converts Angiotensin II (Ang-II) into Angiotensin 1–7 (Ang 1–7) with anti-inflammatory effects. The chronic ACE-2 downregulation in COVID-19 results in the accumulation of Ang-II, which promotes inflammation, fibrosis, and prothrombotic phenotypes [38,39,40].

The expression of the ACE-2 receptor is ubiquitous, and specifically, it has been shown on type 2 pneumocytes of lung alveoli. The cardiac system, gastrointestinal tract, bile duct, and kidneys also express ACE-2 [41]. Co-expression of ACE-2 and TMPRSS in the kidney was primarily identified in the apical surface of proximal tubule cells and podocytes, and to a lesser extent, in distal tubule cells, collecting duct cells, and glomerular parietal epithelial cells [37,42], suggesting that the kidney may also be an important SARS-CoV-2 target.

### 2.2. The Role of Inflammation and Cytokine Storm

Previous studies suggested that the innate immune system reacts against SARS-CoV-2 directly by recognizing pathogen-associated molecular patterns (PAMPs) with pattern recognition receptors (PRRs) and indirectly through damage-associated molecular patterns (DAMPs) with the local release of cytokine, recruitment of inflammatory cells, and stimulation of the adaptive immune response [43,44,45]. Neutrophils are the first cells recruited on acute inflammation sites and contribute to the local inflammation with neutrophil extracellular traps (NETs). A high blood neutrophil count is correlated with disease severity [46,47] and myeloid-derived suppressor cells, which induce the expansion of regulatory T cells (Treg) and are specifically elevated in COVID-19 [48,49]. In the most severe forms, COVID-19 is characterized by systemic inflammation resulting in multi-organ failure (MOF), acute respiratory distress syndrome (ARDS), and death. Several early studies about COVID-19 reported plasma cytokine levels above the normal range and highlighted the role of the “cytokine storm”, defined as a physiological reaction in which the innate immune system causes an uncontrolled and excessive release of pro-inflammatory cytokines in response to the infection [50]. Innate and adaptive immunity are involved in the genesis of a cytokine storm. Cytokines are small proteins secreted by cells that act as intercellular mediators and regulate inflammatory response [51]. It has been shown that SARS-CoV-2 infected cells have a unique and inappropriate inflammatory response defined by low levels of type I and III interferons associated with the elevated release of neutrophil-macrophage recruiting chemokines [52].

One peculiar example is given by IL-6, a pro-inflammatory cytokine key mediator, active in the acute inflammatory response and implicated in MOF, including AKI. Increased plasma levels of IL-6 have been documented in previous retrospective studies enrolling COVID-19 patients, suggesting a main role of IL-6 in the cytokine storm in COVID-19 [53,54,55]. This becomes very intriguing considering that in clinical practice, there are agents that block either IL-6 cytokine or its receptor, and this may represent a therapeutic opportunity for patients with COVID-19 [56,57,58].

### 2.3. A Link between COVID-19 and Bacterial Sepsis Induced AKI

As an intriguing point, Alexander and colleagues have found similarities between sepsis-associated AKI (S-AKI) and COVID-19-related AKI [19]. The morphological and molecular profiles in both conditions include microvascular dysfunction with inflammation, an intense antiviral response with macrophage dominant cellular phenotype, and T cell response, especially in the tubulo-interstitial space. The inflammation as the predominant driver of COVID-19-related AKI is confirmed at the genomic and proteomic levels. Several studies reported an immunological cell death pathway to restrict viral replication and metabolic reprogramming with mitochondrial dysfunction (decreased oxidative phosphorylation and increase of ceramide signaling) in the kidneys of patients with COVID-19 as well as in S-AKI [59,60,61].

### 2.4. The Role of Endothelial Disruption and Coagulation

The association between NETs and endothelitis has been documented in COVID-19 [62,63]. This mechanism may have an important pathogenic role since it leads to the disruption of the endothelium integrity and the exposition of sub-endothelial collagen and tissue factors such as the von Willebrand factor (vWF), which could trigger a hypercoagulability state [46,64]. The A-disintegrin and metalloprotease with thrombospondin type 1 motif 13 (ADAMTS13) normally cleaves to vWF polymers, and a higher vWF/ADAMTS13 ratio occurs in ICU-admitted and mechanically ventilated COVID-19 patients, hence demonstrating a possible link between COVID-19, inflammation, and thrombosis [65,66,67]. A procoagulative pattern is demonstrated with increased blood levels of coagulation and fibrinolysis activation biomarkers, which forecast a higher incidence of microvascular thrombosis in organs such as the lungs and the kidneys [68].

## 3. Role of Dialysis in the Treatment of Patients with COVID-19 Disease

EC procedures and cytokine inhibitors (i.e., tocilizumab, sarilumab, steroids) have been tested to improve outcomes in COVID-19 patients. Blood purification techniques have been used previously as a strategy to remove inflammatory circulating mediators such as cytokines and DAMPSs in patients with sepsis. They also have been proposed in the recent consensus conference of Acute Disease Quality Initiative [21] as possible therapeutic tools in critically ill COVID-19 patients [31,69,70]. There are four EC techniques that can be used to remove cytokines: hemoperfusion (HP) with adsorbent cartridge, renal replacement therapy (RRT) with adsorptive filters, therapeutic plasma exchange (TPE), and RRT with Medium cut-off (MCO) or High cut-off (HCO) membranes [71,72]. RRT includes intermittent (hemodialysis HD, hemodiafiltration HDF, hemofiltration HF) and continuous procedures (continuous veno-venous hemofiltration CVVH, continuous veno-venous hemodialysis CVVHD, and continuous veno-venous hemodiafiltration CVVHDF). In addition, coupled plasma filtration adsorption (CPFA) will be briefly exposed in this paper as a nonspecific extracorporeal filtration method.

### 3.1. Hemoperfusion

HP can remove inflammatory mediators such as DAMPs and PAMPS and consequently reduces plasma levels of cytokine/chemokine [73]. Blood is purified through the removal of plasma solutes by adsorption into either activated charcoal beads or resin beads contained in an adsorbent cartridge [74,75]. Resins have a greater affinity for lipophilic molecules, while charcoal has a greater affinity for hydrophilic molecules. Hemoperfusion can be used in selected patients with hemodynamic instability and systemic inflammatory syndrome, with high plasma levels of cytokines, to limit systemic damage caused by immune hyperactivation. Several types of hemoperfusion cartridges targeting endotoxins or cytokines are available, and have been used in critically ill COVID-19 patients, such as CytoSorb, oXiris, Biosky filter, SeaStar CLR filter, HA280, and HA330 Jafron [76]. Based on performance tests and clinical experience [77,78,79,80], Cytosorb cartridges received FDA emergency authorization for use in COVID-19 patients [81]. Cytosorb HP consists of a cartridge containing a resin with beads of polyvinylpyrrolidone and it can be added to an extracorporeal circuit pre- or post-dialyzer or configured as a standalone. It has been shown that the Cytosorb cartridge determines an improvement in oxygenation index, a CRP reduction, and hemodynamic stabilization in COVID-19 patients [73,76].

In a multicenter retrospective registry, a CytoSorb membrane was used in 52 COVID-19 patients in parallel with extracorporeal membrane oxygenation (ECMO) and administration of HP therapy followed the indications of FDA: 2 CytoSorb for 12 h each, followed by 2 CytoSorb for 24 h each, and clinical reassessment at 72 h to determine clinical benefit for the continuation of therapy. C-reactive protein, ferritin, and D-Dimer decreased during treatment. In this registry study, which did not enroll a control group, mortality was 17.3% (9/52) at day 30, 26.9% (14/52) at day 90, and 30.8% (16/52) at follow-up (maximal of 153 days) [82]. Another retrospective study of 492 patients treated with two CRRT sessions with Cytosorb displayed a decrease in serum IL6, CRP, lactate dehydrogenase, and SOFA score [83]. According to these results, the CytoSorb membrane may represent an adjuvant therapy for critical COVID-19, thanks to the modulation of the inflammatory response, the improvement of microcirculation, and the consequent preservation of tissue perfusion. Nevertheless, there is no unique point of view on the use of CytoSorb in SARS-CoV-2 infection. In a prospective, randomized controlled pilot study, 50 COVID-19 patients with vasoplegic shock requiring norepinephrine were randomized to receive CVVHD with Cytosorb (23 patients) or CVVHD without an adsorbent cartridge (26 patients). The effects on inflammatory markers, catecholamine requirement, and rate of adverse events were similar between the groups, so no clinical benefit was found in the HP group [84]. To overcome the limitation of conflicting results of multiple small studies, several ongoing larger studies have been started [21]. Among them, particular attention should be reserved for a registry study of Aurora (NCT04391920), including 500 critically ill patients with COVID-19, and another study in Belgium, which includes 24 critically ill patients with COVID-19 and tests the efficacy of CytoSorb against the standard of care. These studies could help to clarify the role of CytoSorb cartridges in severe COVID-19.

The FDA also gave emergency authorization for the clinical use of the oXiris membrane in COVID-19 [85]. The oXiris membrane is a high-flux acrylonitrile 69 (AN69) membrane whose absorptive surface is treated with polyethyleneimine grafted with heparin; AN69 filters have a hydrophilic membrane structure and adsorb proteins like a hemoperfusion column. The ability to remove cytokines, chemokines, endotoxins, and lactate from the blood of AN69 improves the hemodynamic status and systemic perfusion of patients [86,87,88]. Although no randomized controlled trials exploring the efficacy of the oXiris membrane have been carried out, there are numerous case series supporting their use in clinical practice. Previous studies have shown a greater efficacy of the oXiris membrane to remove both cytokines and endotoxins compared to the other two major adsorbent cartridges, namely Toraymyxin and CytoSorb [86,89]. Only a few trials are currently evaluating the oXiris filter in COVID-19 patients. Villa et al. demonstrated that early intervention time with oXiris for cytokine adsorption was correlated with better survival in COVID-19 patients (mortality rates, as calculated by APACHE IV, were 8.3% lower after treatment) and with an improvement of hemodynamic, pulmonary parameters and SOFA score as well [54]. Results from a single-center open-label single-arm study in Northern Macedonia [90] and from a multicenter trial lead in Mexico City comparing CRRT with AN69 vs. oXiris in COVID-19 patients [91] are eagerly expected. Interestingly, the use of Cytosorb and the oXiris cartridge in critically ill COVID-19 patients improves the oxygenation index, reduces CRP, and warrants hemodynamic stabilization in previous observational studies [92,93,94,95,96].

HA330 is a synthetic resin hemofilter composed of polystyrene divinylbenzene copolymers specifically developed to remove cytokines from the blood in patients with sepsis or endotoxemia [97]. HA330 showed favorable outcomes in acute respiratory distress syndrome in terms of oxygenation, reduction of lung edema, and circulating and alveolar cytokine levels in patients with sepsis shock and acute respiratory distress syndrome (ARDS) [98]. Specifically, in patients with COVID-19, a single-center prospective cohort study demonstrated that the early use of HA-330 hemoperfusion in addition to standard therapy improves organ failure outcomes (SOFA score, mechanical ventilator-free day) and may indirectly reduce the mortality rate [99]. The limited sample size of this study and the lack of confirmation studies in COVID-19 patients complicate the interpretation of these results.

The severity of COVID-19 can also be affected by superimposed infections, which frequently occur in COVID-19 patients during long intensive care unit (ICU) stays [100,101,102]. Gram-negative bacteria are the most isolated bacteria in COVID-19 patients [103,104,105]. An international, prospective, observational web-based study (EUPHAS2 registry) reported that the use of polymyxin B-immobilized polystyrene column direct hemoperfusion (PMX-DHP) for two consecutive days in COVID-19 patients provides effective endotoxin adsorption and it is associated with hemodynamic stabilization [106]. Polymyxin B-immobilized polystyrene column is a medical device that uses the antibiotic polymyxin B to bind and neutralize lipid A, the active center of endotoxin in patients with COVID-19 and concomitant bacterial infections. As shown in previous studies [57,106], the use of PMX-DHP could be performed in selected patients with endotoxic shock unresponsive to standard treatment. Cytokine measurement pre-and post-PMX-DHP revealed decreased levels of IL-6 with a relatively high risk of circuit coagulation, probably related to septic shock.

### 3.2. RRT with Absorptive Filters

Early introduction of RRT in patients with AKI and COVID-19 shows benefits that include adjustment of the acid base, electrolytes, and water balance. The use of a cytokine-absorbing hemofilter allows an increase in benefits through the removal of inflammatory mediators. In addition, COVID-19 patients with end-stage renal disease (ESRD) undergoing chronic hemodialysis may receive beneficial effects in switching from their standard regular hemofilter to a cytokine-absorbing hemofilter. Polymethyl methacrylate (PMMA) membrane on hemodiafiltration is highly effective in the treatment of severe sepsis due to the removal of many kinds of cytokines from the blood. The PMMA membrane does not activate complement and allows the removal of medium-high molecular weight molecules (IL-6, IL-8 e, HMGB-1) via the convection/diffusion mechanism. The clinical trial Dial-COVID (NCT05040737) testing PMMA and polysulfone membranes in COVID-10019 dialysis patients is ongoing [107].

Absorptive characteristics of the AN69 membrane (oXiris) have been exposed above.

### 3.3. Therapeutic Plasma Exchange

In the TPE process, whole blood is separated into cellular components (erythrocytes, leucocytes, platelets) and plasma; the latter is discarded and replaced with a plasma substitute, mainly albumin and fresh frozen plasma [108]. TPE has been proposed for the treatment of COVID-19 disease [109,110] thanks to its ability in the removal of inflammatory cytokines, such as IFNγ, IL-3, IL-10, IL-1B, IL-6, IL-8, and TNFα) [111,112,113,114]. The theoretical benefits of TPE in COVID-19 are questionable because it removes not only cytokine but other plasma components such as proteins, anti-inflammatory mediators, immunoglobulins, and complement, which can protect against secondary infections [115,116,117]. A randomized controlled clinical trial performed in critically ill COVID-19 patients suggests that TPE plus standard treatment (i.e., dexamethasone, anticoagulant, ribavirin) could be a safe adjunct therapy in critically ill COVID-19 patients with faster clinical recovery and no increased 35-day mortality. In fact, a trend of decreased mortality risk (20.9% vs. 34.1%) was demonstrated in patients treated with TPE compared to the control group, although this difference was not statistically significant. Nevertheless, the combined treatment with TPE was associated with less time on mechanical ventilation (MV) and a shorter ICU length of stay compared with the controls. Moreover, in the intervention group (standard therapy plus TPE), apart from the reduction in all inflammatory biomarkers, a correction of COVID-19-associated thrombus inflammation markers was reported. In particular, it has shown an increase in ADAM-13 activity along with a significant decrease in D-dimer and IL-6 [118]. Several trials have been started testing the use of TPE in COVID-19 patients [119], but available data provided conflicting results due to, at least in part, patient selection bias.

TPE may be indicated in patients with severe COVID-19 and a pathological inflammatory response with cytokine release syndrome; early initiation of TPE prior to end-organ reduces toxic cytokines, corrects coagulopathy, and removes viral particles, therefore improving clinical outcomes [120]. The positive results in terms of lower mortality among patients receiving TPE could be related to the lower severity of COVID-19 rather than the true TPE effects [121]. Hence, the results of ongoing trials are eagerly expected to clarify the real impact of TPE on COVID-19 patients.

### 3.4. Medium Cut-Off (MCO) Membrane and High Cut-Off (HCO) Membrane

Some of the most recent developments in membrane technology for RRT are represented by MCO and HCO membranes. Because of their wider pore size, they are characterized by a significantly increased sieving curve compared to standard high-flux (HF) membranes. Middle molecular weight molecules, including cytokines in the range of 20 kDa to 50 kDa, are successfully removed both with MCO and HCO hemofilters by means of diffusion and convection. The difference between MCO and HCO membranes resides in a narrower range of pore size (mean pore radius is 5 nm and 10 nm, respectively), providing a more selective remotion of solutes with negligible serum albumin losses when using MCO routinely compared to HCO filters [122]. Previous trials have documented a reduction in both plasma levels of inflammatory cytokines and transcription of pro-inflammatory cytokines due to the enhanced removal of soluble mediators with MCO membranes [123]. Moreover, a reduction in serum concentration of free light chains (FLC) and inhibition of leukocyte chemotaxis have been reported [124]. Following these pieces of evidence, Ronco et al. have investigated the rationale for using MCO in COVID-19 patients undergoing chronic hemodialysis. The long-term reduction in the concentration of cytokines, large uremic toxins, and FLC with MCO membranes may prevent severe presentations of SARS-CoV-2 disease in patients with dialysis-dependent ESRD [125]. However, further studies are needed to clarify the role of these membranes in COVID-19 patients.

### 3.5. Nonspecific Removal of Protein

CPFA is a nonspecific extracorporeal filtration method, which separates cellular components from plasma; the latter flows into a sorbent cartridge of styrene resin and then returns into the blood. The hydrophobic resin cartridge acts as a nonspecific removal of inflammatory mediators without losing proteins like albumin [126,127,128]. In vitro experiments demonstrated a better binding of cytokine in plasma filtration compared with HP due to the lower flow rate of plasma into the cartridge and the longer time of contact [129]. Few data are available on the use of CPFA in COVID-19 patients, and larger randomized studies are needed. Ciftici et al. reported the clinical course of two COVID-19 patients who underwent the use of CPFA and CRRT and experimented with a reduction of IL-6 and D-dimer [127]. However, clinical studies enrolling patients with sepsis have not shown encouraging results (even with increased mortality risk!), so this technique was not further evaluated for this aim.

### 3.6. Disadvantages of Using Extracorporeal Treatments

Despite potential benefits derived from the use of the EC techniques, disadvantages need to be mentioned. First, EC treatment requires adequate vascular access (a central venous catheter or CVC) to allow blood to flow through the EC machine circuit and the filter or cartridge. The application of CVC is accompanied by the risk of complications during the insertion procedure, including heart arrhythmias, artery puncture, vein perforation, bleeding and hematoma, and pneumothorax [130]. Prolonged use of CVC poses a greater risk of colonization of the tip from resident flora of the skin at the insertion site, leading to CVC-related bloodstream infection (CVC-BSI) and sepsis with multi-organ failure, increasing ICU stay and mortality in COVID-19 patients. Moreover, catheter dysfunction, due to either mechanical problems (kinking, malposition) or thrombotic complications (intracatheter and pericatheter thrombosis, fibrin sheath formation), catheter adherence to the vessel wall, and central venous stenosis (CVS) can be observed [131].

Second, there is no shortage of disadvantages related to the EC technique itself. The most common complications of HP include arrhythmia, thrombocytopenia, and increased bleeding risk with coagulation disorders [132]. TPE is associated with the risk of hypocalcemia due to the use of citrate as an anticoagulant, which chelates calcium; the risk of transfusion reactions due to replacement fluid (most commonly, hypotension when albumin is used, and urticaria when plasma is used); and significant removal of coagulation factors, medication, endogenous immunoglobulins, anti-inflammatory mediators, and therapeutic monoclonal antibodies [133]. As stated above, HCO membranes are associated with significant serum albumin losses, that are mitigated with the use of MCO membranes [122].

Third, EC-related disadvantages are not obviously balanced by clear advantages. The clinical outcome of patients does not seem to be improved by EC cytokine removal due to still unclear pathophysiology, nonspecific cytokine removal, and scarcity of information related to the timing and dose of treatments.

Moreover, when a patient is directed to an EC technique, multiple sessions should be scheduled in subsequent days. The high-cost burden and too sophisticated and complicated techniques may limit the use of EC treatment in many centers; therefore, providing specific guidelines to lead the correct prescription of EC techniques in COVID-19 patients is of utmost importance to limit costs and waste.

## 4. Conclusions

As summarized in this review and as highlighted in other articles, acute kidney injury is a multifactorial condition and a common complication of COVID-19 [3,4,5,6,7], associated with an increased mortality rate (54.2%) among hospitalized patients with SARS-CoV-2 infection [134].

The common opinion is that despite the evolution of blood purification techniques together with pharmacology therapies and the deeper acknowledgment of SARS-CoV-2 disease, the management of patients with AKI and COVID-19 remains challenging. Table 1 summarizes some of the studies related to the use of TPE, CytoSorb, oXiris membrane and PMX-HP in COVID-19 patients. Current studies about EC techniques in patients with AKI and COVID-19 suffer from several limitations, such as the small sample size, heterogeneous patient populations, and the different time points at which patients have been studied during the course of the pandemic. Moreover, evidence of the definite clinical advantage of using EC techniques is controversial, and results from large prospective trials are needed. As COVID-19 is still ongoing, the importance of continuing the analysis of the efficacy and safety of these therapeutic tools is crucial in order to provide a prompt and appropriate treatment to COVID-19 patients with AKI in the near future. Future aims of clinical research around this topic should also include a measurable definition of the cytokine storm, a definition of biomarkers that can guide more appropriate EC treatment, a definition of the dose and timing to minimize EC circuit-related adverse effects, minimizing albumin loss and drug removal, and increasing the effect of cytokine removal on patient’s hemodynamics.

## Figures and Tables

**Table 1:** Studies related to the use of TPE, CytoSorb, oXiris membrane and PMX-HP in COVID-19 patients.

Study Authors, EC Technique	Type of Study	Sample Size	Treatment	Main Results
Faqihi et al., 2021 [118] TPE	Single-center, open-label, randomized clinical study	87 (43 in the intervention group)	Standard empirical therapeutic regimen (antivirals, antibiotics, dexamethasone, anticoagulants and ICU supportive care) plus TPE	Better clinical recovery and less time on MV and ICU length stay compared to controls, along with no increased 35-day mortality.
Rampino et al., 2020 [135] CytoSorb	Observational study	9	Patients were treated with Cytosorb (2 consecutive sessions)	IL-6, IL-8, and TNF-α decreased after HP. Improved survival (80% in TG vs. 0% in CG). Intubation required less frequently (40% in TG vs. 100% CG).
Tea Song et al., 2021 [82] CytoSorb HP	Multicenter observational study	52	Patients received veno-venous ECMO plus CytoSorb therapy	A reduction in the incidence of mortality: 17.3% (9/52) at day 30, 26.9% (14/52) at day 90 and 30.8% (16/52) at follow-up (153 days). C-reactive protein, ferritin, and D-Dimer decreased during treatment.
Yatin Mehta et al., 2021 [92] CytoSorb HP	Case series	3	Single CytoSorb device plus tocilizumab, antivirals, hydroxychloroquine, azithromycin	C-reactive protein levels decreased by 91.5%, 97.4%, and 55.75 %, and mean arterial pressure improved by 18%, 23%, and 17 % in patients 1, 2, and 3, respectively, on day 7 post-therapy.
Peng J et al., 2022 [93] CytoSorb HP	Case series	10	Patients received 2 HP, median CytoSorb perfusion time of 47 h	The level of IL-6 significantly decreased after treatments (712.6 vs. 136.7 pg/mL, *p* = 0.005). Improvement of PaO2/FiO2 (118 vs. 163 mmHg, *p* = 0.04) and lactate levels (2.5 vs. 1.7 mmol/L, *p* = 0.009). Hemodynamics measured by norepinephrine/MAP ratio slightly improved after treatment (17 [0–68] vs. 8 [0–39] µg/h/mm Hg, *p* = 0.09). Albumin mildly decreased after CytoSorb. No significant changes were found in red blood cell counts, white cell counts, and platelets.
Nassiri et al., 2021 [94] Cytosorb HP	Case series	26	Patients received 2 hemoadsorption treatments with CytoSorb	PaO2/FiO2 ratio improved significantly. 19 patients reached P/F ratio above 250 mmHg post intervention. Non-survivors improved to the same degree as survivors, except for their CRP levels. Patients stayed on the ICU for 9 days and 21 of them survived.
Stockmann et al., 2022 [84] Cytosorb HP	Prospective, randomized controlled pilot study.	50	A total of 23 were randomized to receive CytoSorb and 26 patients to receive standard of care	Resolution of vasoplegic shock was observed in 56.5% of patients in the CytoSorb and in 46.2% of patients in the control group after a median of 5 days. Mortality rate was respectively 78% and 73%. The effects on inflammatory markers, catecholamine requirements, and the type and rates of adverse events were similar between the groups.
Alharthy et al., 2021 [83] Cytosorb HP	Retrospective study	492	A total of 2 ± 1 CRRT sessions with CytoSorb	Decreased SOFA scores, lactate dehydrogenase, ferritin, D-dimers, C-reactive protein, and interleukin-6; increased PaO2/FiO2 ratio, and lymphocyte counts (all *p* < 0.05).
Berlot et al., 2022 [136] Cytosorb HP	Retrospective study	4	Two patients who received TCZ alone (CG) and in others, two in whom it was associated Cytosorb (TG)	IL-6 increased in TG, CRP decreased in all patients; the PaO2/FiO2 increased in three patients. All four patients were weaned from mechanical ventilation
Zhang et al., 2020 [95] oXiris HP	Case series	5	CRRT with oXiris membrane	CRP, IL6, IL10, APACHE II and SOFA decreased after treatment; PaO2/FiO2 increased
Kang et al., 2022 [137] oXiris HP	Case series	17	Patients in the intervention group immediately received CRRT with oXiris filter plus conventional treatment, while those in the control group only received conventional treatment.	No significant difference between the two groups in terms of cytokine storm
Rosalia et al., 2022 [138] oXiris HP	Prospective Cohort Study	44	All patients were treated with ≥1 cycle of (CVVH) with oXyris; of these, 30 severe patients received CVVH within 4–12. Another 14 patients admitted with mild-to-moderate symptoms progressed to severe disease and were placed on EBP during hospitalization	Decrease in CRP, and control of IL-6 and procalcitonin
Villa et al., 2020 [54] oXiris HP	Prospective study	37	CRRT with oXiris	IL-6 level reduction, attenuation of systemic inflammation, multi-organ dysfunction improvement, and reduction in expected ICU mortality rate.
Spencer et al., 2020 [96] oXiris HP	Case series	2	Patients treated for 48 h with an oXiris filter exchange at 24 h.	Rapid improvements in oxygenation
Ugurov et al., 2020 [139] oXiris HP	Case series	15	Respiratory support, extracorporeal blood purification using oXiris, 300 U/kg heparin to maintain activation clotting time ≥ 180 s.	Increase of thrombocytes and white blood cells, stable levels of IL-6 (<50 ng/mL), and a decrease in CRP and fibrinogen.
Padala et al., 2020 [56] oXiris HP	Case series	3	CVVHDF and oXiris	Decreased levels of inflammatory markers including interleukin-6 (IL-6), erythrocyte sedimentation rate (ESR), and C-reactive protein (CRP).
De Rosa et al., 2020 [106] PMX-HP	Prospective and observational web-based database (EUPHAS2 registry)	12	Patients received PMX-HP, and 75% need CRRT	SOFA score progressively improved over the next 120 h—decrease of median endotoxin activity assay (EAA), improvement of hemodynamics.
Kataghiri et al., 2020 [57] PMX-HP	Case series	12	Patients treated with PMX-DHP on two consecutive days each during hospitalization.	High risk of circuit coagulation

Abbreviations: HP hemoperfusion, CG control group, CRP C-reactive protein, CRRT continuous renal replacement therapy, CVVHD continuous veno-venous hemofiltration, CVVHDF continuous veno-venous hemodiafiltration, EC extracorporeal, ECMO extracorporeal membrane oxygenation, PMX-HP polymyxin B hemoperfusion, TCZ Tocilizumab, TG treatment group, TPE therapeutic plasma exchange.

## Data Availability

Not applicable.
